# Primary Aneurysm of the Medial Marginal Vein of the Foot

**DOI:** 10.1155/2015/374691

**Published:** 2015-10-21

**Authors:** D. Casian, V. Culiuc

**Affiliations:** Department of General Surgery, University of Medicine and Pharmacy “Nicolae Testemitanu”, 2008 Chisinau, Moldova

## Abstract

The primary superficial venous aneurysms of the foot are very rare. A 34-year-old female patient developed a dorsal foot mass during the second trimester of pregnancy with no history of previous trauma, puncture, or infection. One year later, she was referred to the surgical department for excision of “foot hygroma.” Based on the clinical findings, the venous aneurysm was suspected and duplex ultrasound confirmed the diagnosis of the aneurysm of the medial marginal vein of the foot. Excision of aneurysm with bipolar ligation of marginal vein was performed under local anesthesia. The postoperative evolution was uneventful. The authors hope that the presented case report will increase the awareness of general practitioners, dermatologists, and surgeons regarding the superficial venous aneurysms of lower limbs.

## 1. Introduction

During the last decades, the aneurysms of the superficial venous system become diagnosed and described more frequently because of the wide implementation of vascular ultrasound. The main trunks of the great or small saphenous veins are affected most typically as a consequence of pathologic venous reflux and degenerative changes of the venous wall in patients with varicose veins. Superficial venous aneurysms of the upper extremities (cephalic and basilica veins) and neck (external jugular vein) were reported sporadically [1–3]. Only several case reports were published in the literature regarding superficial venous aneurysms located on the dorsum of the foot [4–6]. We present the case of primary aneurysm of the medial marginal vein of the foot developed during the pregnancy.

## 2. Case Presentation

A 34-year-old female presented to the department of general surgery with complaints of unpainful mass, localized on the dorsal surface of the right foot. In out-patient clinic, she was examined by a general practitioner who established the diagnosis “foot hygroma” and referred her to the hospital for surgical excision. The patient noted for the first time a mass over her right foot during the second trimester of pregnancy with slow progressive enlargement of the tumor over the course of a year. Her recent medical history was unremarkable with no events of blunt trauma, puncture or any infectious process in the affected region of the right foot.

**Figure 1:**
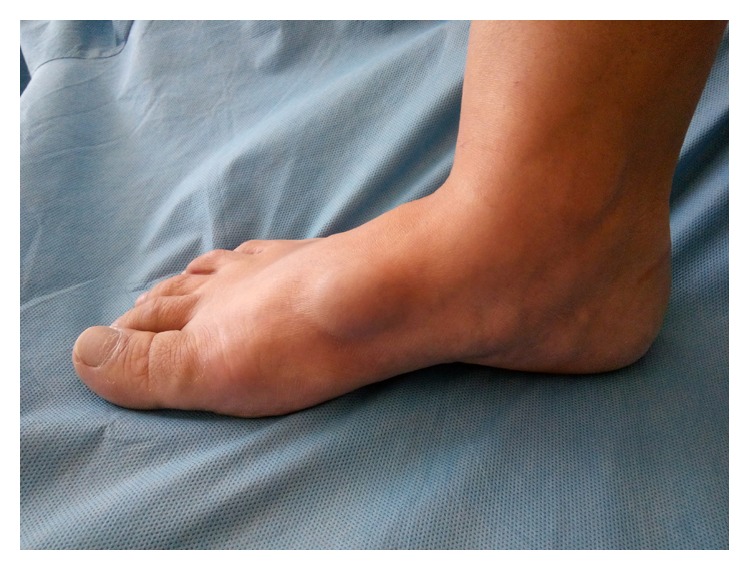
Appearance of a venous aneurysm of the medial marginal vein of the foot initially diagnosed as a foot hygroma (patient is in upright position).

At admission to the department, inspection revealed the nontender, round, elastic, slightly mobile mass with a size 30 × 20 mm located subcutaneously on the dorsal surface of the right foot (Figure 1).

**Figure 2:**
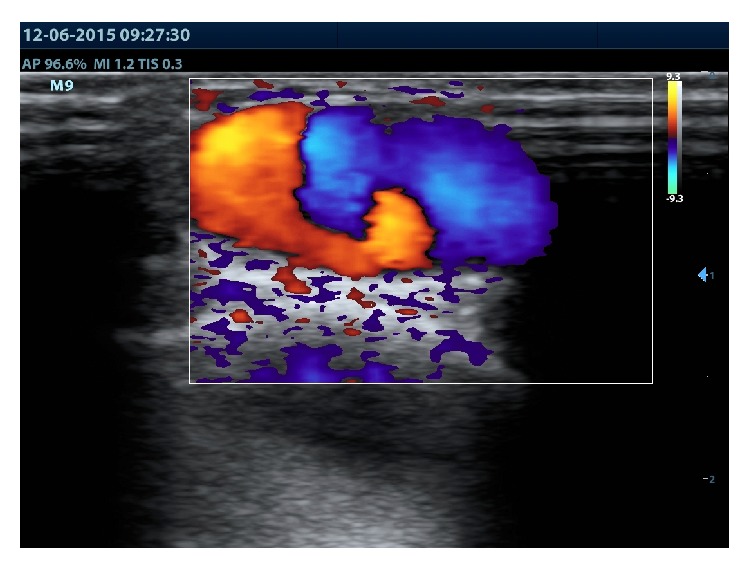
Turbulent flow inside the sac of aneurysm (“yin-yang” sign) detected by duplex ultrasound during the compression of venous foot pump.

Palpation of the mass has not demonstrated any pulsation or thrill and there was no bruit on auscultation. Attempts to compress the mass against bone with patient upright resulted in only minimal decrease of tumor size whereas in recumbent position of the patient with elevated leg the fast spontaneous collapse of the tumor was observed. Based on clinical findings, the aneurysm of the superficial vein of the foot was suspected and Color Doppler and duplex ultrasound were performed to confirm the diagnosis. Duplex scanning showed the ovoid anechoic dilatation of the medial marginal vein of the foot measuring 2.5 cm in diameter and extending over a length of 3.5 cm with no thrombus inside. There was no detectable spontaneous flow in the lumen of the aneurysm with appearance of a turbulent to and fro flow (the so-called “yin-yang” sign) during the compression of the venous foot pump (Figure 2).

The terminal and subterminal valves of the saphenofemoral junction and all valves along the course of great saphenous vein were competent. The scanning of the arterial and venous systems of both lower limbs did not show any other abnormalities.

**Figure 3:**
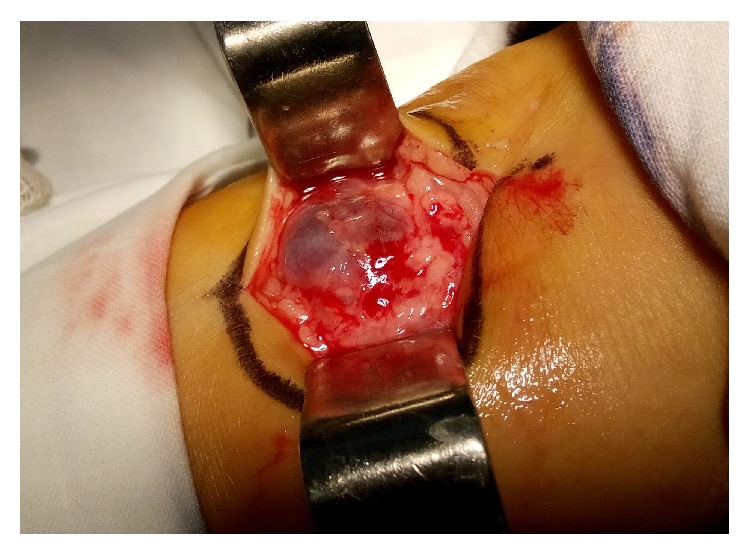
Intraoperative aspect of the venous aneurysm before excision.

The patient was operated under local tumescent anesthesia with 60 mL of 0.1% lidocaine solution. Via 5.0 cm longitudinal skin incision, the sac of aneurysm was visualized and has been dissected from the surrounding tissues (Figure 3).

Excision of the aneurysm was performed after bipolar ligation of marginal vein (diameter of vein at both sides of the aneurysm: approximately 2.0 mm) and wound was closed with absorbable running intracuticular suture. The foot and leg were wrapped with elastic bandage and after 6 hours the patient was discharged. The postoperative evolution was uneventful with moderate edema of the foot which resolved spontaneously to the second postoperative day.

Histopathological examination of the sac of aneurysm revealed normal but thinned vein wall with reduced number of smooth muscle cells. At one-month follow-up, there were no signs of recurrence and the patient has declared complete satisfaction by outcome of intervention.

## 3. Discussion

The superficial venous aneurysms are usually benign pathological conditions with asymptomatic course although the development of thrombus in the lumen of aneurysm can be associated with local inflammatory reaction and pain. There is also potential risk of significant bleeding in a case of traumatic injury of aneurysm. Keshelava et al. [7] described two cases of proximal great saphenous vein aneurysm that resulted in pulmonary embolism.

Because of its rarity, superficial venous aneurysms are frequently misdiagnosed by general practitioners and surgeons as lipomas or other soft tissue tumors. In our case, the patient was initially diagnosed with foot hygroma. Difficulty in the squeezing of the aneurysm determined during the examination of the patient in orthostatic position may be, at least theoretically, responsible for this erroneous diagnosis. Limited compressibility of a venous aneurysm is usually caused by the thrombosis of aneurysmal sac. It was not a case in our patient, so we can suppose that a minimal compressibility during standing was caused by relative high pressure inside of the aneurysm transmitted from the plantar venous plexus of Lejar to the medial marginal vein via perforators: vein of the first metatarsal space and medial foot perforator veins. It should be kept in mind that if a pathological mass is diagnosed at the level of the lower limb, it is always useful to examine the patient in both positions, upright and recumbent with elevated leg. The lump collapse with leg elevation is a pathognomonic sign of nonthrombosed venous dilatation.

In contrast to secondary venous aneurysm, which usually develops due to venous hypertension, trauma, or inflammation, the etiology of primary venous aneurisms is not completely understood. It is interesting to note that all the published case reports of dorsal foot venous aneurysms describe this finding exclusively in young female patients. The impact of hormonal changes upon aneurysm development should therefore be suspected. In our case, the aneurysm starts to grow during pregnancy, a specific feature of vascular malformations. Burnley et al. [8] also reported the case of a dissecting superficial venous aneurysm in the branch of small saphenous vein that appeared during the third trimester of pregnancy. The authors believe that haemodynamic changes characteristic for pregnancy, increased blood volume and cardiac output, can be responsible for focal venous dilatation. In another case report, the etiology of dorsal foot venous aneurysm was attributed by authors to the trauma caused by repeated compression of the vein with sandal straps [6]. However, this original theory can not explain the unilateral development of the aneurysm in that patient. We suppose that it is more correct to consider the primary venous aneurysm, either superficial or deep, as a venous malformation. According to the modified Hamburg classification of congenital venous malformations, venous aneurysms refer to truncular localized dilated lesions. Truncular lesions do not have the embryonic characteristics of mesenchymal cells as observed in the extratruncular lesions and hence do not possess the evolutional ability to proliferate [9]. The risk of recurrence after treatment of venous aneurysms is minimal to none, this fact being confirmed in all published cases.

Treatment of superficial venous aneurysm is usually indicated due to esthetic considerations; however, the compression of the radial nerve that resulted in wrist pain and sensorial disorders was described in the case of aneurysms situated on the upper limbs [1, 10]. Furthermore, turbulent flow inside the sac of the aneurysm predisposes to the development of thrombosis. Two curative approaches for superficial venous aneurysms of the foot are described in the literature: sclerotherapy and excision. Although some authors report excellent clinical and esthetic outcome using sclerotherapy [3, 4], in the majority of cases the aneurysms are excised surgically. We support the later approach due to relative simplicity and high efficacy of excision. Sclerotherapy can lead to the sac thrombosis which potentially can extend to the saphenous trunk. Moreover, excision of the aneurysm allows the histopathological examination of the specimen.

In conclusion, primary aneurysm of the medial marginal vein of the foot is a very rare pathological condition that may be easily misdiagnosed as a soft tissue tumor. Careful clinical examination in different positions of the patient, followed by duplex ultrasound, will lead to correct diagnosis and treatment. The authors hope that the presented case report will increase the awareness of general practitioners, dermatologists, and surgeons regarding the superficial venous aneurysms of lower limbs. Further investigations are required to improve our understanding of the etiology and pathogenesis of this disease.

## References

[1] Cakici M., Ersoy O., Ince I., Kiziltepe U. (2014). Unusual localization of a primary superficial venous aneurysm: a case report. *Phlebology*.

[2] Katsoulis I. E., Jader S., Bradpiece H. A. (2003). Primary aneurysm of the basilic vein. *Surgeon*.

[3] Seo S.-H., Kim M.-B., Kwon K.-S., Kim C.-W., Oh C.-K. (2008). Primary venous aneurysms of the superficial venous system. *Angiology*.

[4] Rabe E., Rabe P. (1990). Venous aneurysms in the foot region. *Zeitschrift für Hautkrankheiten*.

[5] Pozarny E., Grandi R., Lane G. (2001). Venous aneurysm of the dorsal venous arch. *Journal of the American Podiatric Medical Association*.

[6] Sadr A. H., Paes T. R. F. (2010). Venous aneurysm of the dorsal venous arch: a case with an unusual etiology. *Journal of the American Podiatric Medical Association*.

[7] Keshelava G., Beselia K., Nachkepia M., Chedia S., Janashia G., Nuralidze K. (2011). Surgical treatment of the great saphenous vein aneurysm resulting in pulmonary embolization in two patients. *Annals of Vascular Surgery*.

[8] Burnley H. M., McCormick D., Hurren J., Gallagher P. J. (2003). Primary venous dissecting aneurysm arising during pregnancy: a case report and review of the literature. *Journal of Clinical Pathology*.

[9] Lee B. B., Laredo J., Lee T. S., Huh S., Neville R. (2007). Terminology and classification of congenital vascular malformations. *Phlebology*.

[10] Kassabian E., Coppin T., Combes M., Julia P., Fabiani J.-N. (2003). Radial nerve compression by a large cephalic vein aneurysm: case report. *Journal of Vascular Surgery*.

